# AI Trustworthiness in the Perioperative Period for Patients with Serious Illness: Scoping Narrative Review

**DOI:** 10.2196/89936

**Published:** 2026-10-01

**Authors:** Bhagvat J Maheta, Rachel L Ross, Isabella Raspi, Christina Keny, Marti N Okech, Selen Bozkurt, Karleen F Giannitrapani

**Affiliations:** 1 Department of Surgery, Northwestern University, 420 E Superior St, Chicago, IL, 60611, United States, 1 312-503-8194; 2 VA Palo Alto Health Care System, Palo Alto, CA, United States; 3 Department of Primary Care and Population Health, Stanford University, 3180 Porter Drive, Palo Alto, CA, 94305, United States; 4 Department of Psychiatry and Behavioral Sciences, Stanford University, Stanford, CA, United States; 5 School of Nursing, University of California, , San Francisco, CA; 6 Department of Biomedical Informatics, Emory University School of Medicine, GA, United States

**Keywords:** AI, surgery, perioperative, systematic review

## Abstract

**Background:**

Despite the promising potential of AI in the perioperative context, the rapid pace of development and diverse implementation warrant a thorough review to consolidate existing knowledge, identify gaps, and assess the use of trustworthiness principles in AI integration into the perioperative period for patients with serious illness.

**Objective:**

The purpose of this study was to address deficiencies in the perioperative AI literature by elucidating the extent to which discussions of equity, ethics, and safety are incorporated, thereby establishing a foundation for the development of robust ethical guidelines for the safe and effective integration of AI in health care.

**Methods:**

We searched PubMed, Embase, CENTRAL (Cochrane Central Register of Controlled Trials), and Scopus for studies published from 2010 to July 2024. We included studies that reported patient functional outcomes, occurred in the perioperative period (30 d before and up to 90 d after surgery), incorporated AI integration, and included patients with serious illness (defined as malignancy, advanced organ failure, frailty, dementia or neurodegenerative disease, or stroke). To ensure reliability and minimize bias, 2 independent reviewers screened all studies at the title or abstract and full-text stages; conflicts were resolved through team consensus. The abstraction form was developed iteratively and was tested through pilot abstractions. Any discrepancies identified during data extraction were resolved through discussion and consensus among the reviewers. The ROBINS-I (Risk of Bias Tool in Nonrandomized Studies of Interventions) tool was used to assess quality. Abstraction and risk assessment were conducted through a blinded, independent dual-review process. A narrative review was compiled from the identified studies.

**Results:**

Of the 10,980 papers identified through the database searches, this review yielded 81 papers that met the inclusion criteria. Analysis of AI implementation strategies revealed foundational efforts toward equitable access, with 6 studies providing open-access tools and several more designing models with simple inputs suitable for low-resource settings (17 studies). Seven studies mentioned their commitment to transparency (eg, publishing code) to enhance safety and trust. However, significant ethical deficiencies persist, particularly regarding input data, as only 2 studies explicitly addressed racial or ethnic disparities, and concerns about lack of sample diversity (16 studies) and the omission of socially relevant features (5 studies) were frequently noted as limitations.

**Conclusions:**

Machine learning for predictive analytics and other types of AI tools for surgical outcomes offer significant potential but require adherence to trustworthiness and safety principles to be clinically viable. Future research should prioritize adherence to guidelines for equity, ethics, and safety, conduct prospective studies, incorporate more external validation of AI models, and facilitate transparent monitoring and reporting of model performance to build clinician and patient trust and encourage broader health care system adoption.

## Introduction

### Rationale

In the perioperative period, patients with serious illnesses such as malignancies, organ failure, or frailty face a diminished quality of life, and this can be exacerbated during the perioperative period, when patients face additional morbidity and mortality [1-3]. However, AI offers a transformative approach to address these challenges in the perioperative period [4]. By analyzing complex patient data, AI—specifically through methods like machine learning (ML), natural language processing (NLP), and artificial neural networks (ANNs)—can predict risks, optimize treatment plans, and assist in critical surgical decision-making and risk stratification [5]. This technology not only aids clinicians but also holds potential for providing personalized patient support and education [6]. Although these AI tools are already becoming integral to perioperative care, the integration of generative AI is still in its nascent stages.

Although the potential benefits of AI are significant, its integration into health care, particularly for vulnerable patients in the perioperative period, introduces crucial ethical considerations. The core ethical issues include data privacy—ensuring that sensitive patient information remains secure—and algorithmic bias, which can perpetuate and even amplify existing health disparities if the data used to train the AI are not representative [7]. There are also concerns about overreliance on technology, where clinicians may defer too much to AI recommendations without exercising their own critical judgment. The Department of Health and Human Services (HHS) characterizes AI trustworthiness into 3 categories: ethics, equity, and safety [8]. The American Medical Association’s (AMA) 2025 governance framework expands trustworthiness by mandating explainable AI tools whose decision logic clinicians can interpret and communicate to patients more transparently [9,10]. The World Health Organization (WHO) emphasizes that trustworthy AI in health care must protect human autonomy, promote safety and well-being, ensure transparency and explainability, foster accountability, advance inclusiveness and equity, and uphold sustainability across the entire AI lifecycle [11]. This is especially important in the perioperative period for seriously ill patients, where the stakes are high, and decisions may need to be made rapidly [12]. A standard for AI ethics in the perioperative period, when patients are highly vulnerable, does not yet exist. It is imperative to establish a robust and appropriate standard to ensure the well-being and safety of patients and to uphold ethical integrity.

The rapid development and diverse implementation of AI in perioperative care have created a compelling need for a thorough review to consolidate existing knowledge, identify critical gaps, and assess the effectiveness and limitations of integrating perioperative AI for patients with serious illness. Although a growing body of literature validates the performance of various AI models, a significant gap remains in understanding the practical strategies for integrating these validated models into clinical workflows to tangibly enhance efficiency and patient outcomes [13,14]. Although there has been work conducted on perioperative AI integration, most of it has not focused on serious illness, and there is still more to uncover related to the ethical implementation of AI in these spaces [14]. Furthermore, there is a distinct lack of research dedicated to the crucial ethical and safety considerations of AI, as well as an exploration of how AI-enabled team augmentation is currently incorporated into the care environment. Anchoring the concept of perioperative AI trustworthiness within standard guidelines, such as HHS and WHO, reinforces the notion that surgical AI systems must not only achieve technical excellence but also demonstrate compliance with internationally accepted principles of ethical design, governance, and continuous quality assurance.

### Objectives

The purpose of this study is to address these deficiencies by systematically reviewing the literature to better elucidate the extent to which current publications incorporate equity, ethics, and safety discussions. This research also aims to provide a comprehensive understanding of the current landscape of AI in perioperative care, establish a foundation for future research, and contribute to informing the development of robust, ethical guidelines to ensure the safe and effective integration of AI in health care.

## Methods

### Protocol and Registration

Our review adhered to the Preferred Reporting Items for Systematic Reviews and Meta-Analysis Protocols (PRISMA) Statement and EQUATOR (Enhancing the Quality and Transparency of Health Research) guidelines [15,16]. We registered our protocol with the PROSPERO (International Prospective Register of Systematic Reviews) database under the registration number CRD42024608387 [17].

### Eligibility Criteria

This review included peer-reviewed publications from 2010 to July 2024 that met the following criteria, organized by the population, intervention, comparator, outcomes, timing, and setting (PICOTS) framework (Table 1). *Inclusion criteria* were patients with serious illness over the age of 18, integration of AI in the perioperative period, reporting any patient quality outcomes, and being peer-reviewed and published in English (for feasibility). Studies that solely focused on radiology or pathology data were excluded from this study, as AI in these fields has been well characterized [18,19]. Studies that only used logistic regression, which is a traditional statistical modeling approach, were also uniformly excluded from this study for feasibility. Serious illness, as operationalized in this research, included the following categories: malignancy, advanced organ failure (eg, end-stage cardiac, pulmonary, renal, or hepatic disease), frailty with dementia or neurodegenerative disease (progressive, incurable conditions with functional decline, such as dementia or Parkinson disease), and stroke with significant residual impairment [1,2]. AI is defined as the field encompassing machine learning and generative AI, enabling systems to learn from data and generate new content, respectively [20-22]. The perioperative period was defined as the period 30 days before and up to 90 days after surgery [23]. Patient quality outcomes were defined as both patient-reported measures of quality of life and surgical factors such as mortality, length of hospital stay, and postoperative complications [24,25]. Conversely, biological intermediaries, such as radiology scans and blood biomarker values, were excluded from the definition of patient quality outcomes [26].

**Table 1.** Population, intervention, comparator, outcomes, timing, and setting (PICOTS) eligibility criteria.

PICOTS	Eligibility criteria
Population	Inclusion Patients with serious illness Adults (18 y and older) Serious illness: Malignancy Any type of malignant cancer. Benign cancers will not be included Advanced organ failure Must be irreversible (unless patient gets an organ transplant) and chronic (no acute processes such as infection) Common examples: end-stage heart failure, COPD^a^, cirrhosis, end-stage renal disease Frailty and dementia or neurodegenerative disease Chronic, irreversible, progressive, and deteriorating conditions with no cure Multiple chronic conditions, significant functional impairments, and an increased risk of mortality Common examples: dementia, Parkinson disease Stroke with significant residual impairment Major stroke and have experienced significant and persistent neurological deficits
Intervention	Inclusion Describe the integration of AI in the perioperative period Exclusion AI is only used for diagnostic radiology
Comparator	N/A^b^
Outcomes	Inclusion Patient quality outcomes reported in the study Patient-reported outcomes relating to quality of life Patient-demonstrated outcomes (eg, arm curl, chair stand, back scratch, chair sit, reach, walk test, overall cognition, memory, etc)
Timing	Interventions with any follow-up period
Study characteristics	Inclusion Peer-reviewed, published manuscript English only (for feasibility)

a COPD: chronic obstructive pulmonary disease.

b N/A: not applicable.

### Information Sources and Search

Our comprehensive literature search encompassed terms related to AI, the perioperative period, and serious illness, as detailed in Multimedia Appendix 1. The search was created through an interactive process using prior published search terms, as well as collaboration with experts in AI (CK, SB). We conducted searches across PubMed, Embase, Scopus, and the Cochrane Central Register of Controlled Trials (CENTRAL) for publications from 2010 to July 2024, yielding an initial 10,980 records, which were reduced to 8942 after removing duplicates.

### Selection of Sources for Evidence

Our study team advisors included a PhD expert in team science, health care systems design, and health services research (KG), a PhD expert in patient engagement, organizational behavior, and health services research (RR), and a PhD and registered nurse expert in AI and the perioperative period (CK). Screening involved 4 co-authors (BM, RR, IR, and MO), each independently reviewing studies at the title or abstract and full-text stages, with reviewers blinded to each other’s assessments. Discrepancies during title or abstract screening were primarily resolved by a designated “gold standard” reviewer (BM or KG), with occasional recourse to majority consensus. At the full-text screening stage, exclusion reasons were systematically recorded, and all conflicts were adjudicated solely by the “gold standard” reviewer (BM or KG). If a study did not specify what percentage of their sample size was diagnosed with malignancy compared to benign pathology, the corresponding author was contacted. If the information was not given within 3 weeks, the study was rejected. Three studies were rejected because of this. Furthermore, any systematic reviews fulfilling the inclusion criteria were used to identify additional relevant studies, which were then incorporated into the title or abstract screening phase. The study selection process, including the number of studies at each stage, was documented using Covidence software to generate a PRISMA flow diagram [27].

### Data Charting Process

The abstraction form was created through an iterative process (Multimedia Appendix 2). This form collected data on participant characteristics, including demographics such as gender, age, and race or ethnicity, as well as the category of serious illness (malignancy, advanced organ failure, frailty or dementia, or stroke). Information regarding the AI intervention was abstracted, including the AI’s purpose in the study (prognostication, screening, decision support, etc), the type of AI used (generative AI or ML), and the specific AI interface or tool. Information related to the validity of the AI model was not abstracted, since the primary objective of this review was to better understand equity, ethics, and safety discussions within the studies, and the focus was on implementation of the AI model rather than its validity. Additionally, the form captured whether the study addressed AI equity, ethics, and safety, and how AI was used within the health care setting, such as a team member or a prompting tool. Data abstraction for the included studies was conducted using Google Sheets, with 2 reviewers per paper. Reviewers (either BM, RR, IR, or CK) independently abstracted each paper, were blinded to each other’s work, and resolved all abstraction conflicts through a consensus discussion.

### Risk of Bias and Quality Assessment

The ROBINS-I tool was used during the data extraction phase to evaluate the quality of each included paper [28], since it allows for risk of bias stratification for nonrandomized studies that examine the effect of an intervention on an outcome. The research team assessed potential biases across the following domains: confounding, selection of participants into the study, classification of interventions, deviations from intended interventions, missing data, measurement of outcomes, and selection of the reported result. This systematic assessment involved 2 independent reviewers (among BM, RR, IR, and CK), who evaluated the risk of bias across multiple domains. Disagreements between the 2 reviewers were resolved through discussion and consensus.

### Synthesis of Results

Given the substantial heterogeneity observed across included studies, stemming from variations in AI implementation, use, and reported patient quality-of-life outcomes, a narrative synthesis of the abstracted data was conducted using a grouping method. We synthesized studies based on malignancy type, AI type, as well as the incorporation of AI equity, ethics, and safety. Studies that included equity, ethics, and/or safety related to AI, as defined in the sections below, were included in this review, and details on these topics were systematically extracted from the manuscripts. The type of AI used was categorized based on whether the individual tools were supervised ML, unsupervised ML, supervised deep learning, unsupervised deep learning, NLP, or linear programming. The definitions used are listed in the sections below. The validity of AI was not considered as part of this analysis. For studies that included AI equity, ethics, and safety, the quotes were extracted and open-coded with dual review until saturation of codes was reached (RR, BM) [29,30]. Then the quotes were consolidated into appropriate themes through open coding and dual review. AI equity, ethics, and safety were defined in the following section.

### Data Items

The following definitions were used to guide the identification and extraction of data related to AI equity, ethics, and safety:

- AI equity: The fair and just distribution of the benefits and opportunities afforded by AI technologies across all populations, regardless of race, ethnicity, gender, socioeconomic status, geographic location, or other social determinants of health, while also mitigating any potential harms or biases that could exacerbate existing health disparities [31]
- AI ethics: Ensuring that the development, implementation, and use of AI adhere to the fundamental ethical principles of beneficence, nonmaleficence, autonomy, justice, and explainability, while mitigating potential risks, such as biased screening data, to promote equitable and trustworthy health care outcomes [31]
- AI safety: The development and implementation of measures and practices that ensure that AI systems used in medical settings are reliable and robust and function as intended, minimizing the risk of harm to patients, clinicians, or the health care system [32]

### Definitions of AI

The following definitions were used to categorize the types of AI identified in the included studies:

- Generative AI focuses on creating new content, such as text, images, or synthetic patient data. It learns the underlying patterns and structures of the data it is trained on and then uses that knowledge to generate entirely new content that is similar in style and characteristics to the training data [33].
- ML algorithms learn patterns from data without explicit programming, enabling them to make predictions or decisions [34].
- NLP focuses on enabling computers to understand, interpret, and generate human language [35].

## Results

### Selection of Sources of Evidence

A total of 10,980 records were considered for review by the research teams. After 2038 (18.6%) papers were removed as duplicates, 8942 were screened at the title or abstract stage. Of 8942 papers, 8687 (97.1%) of those were excluded, and 249 were screened at the full-text stage. Ultimately, 81 studies were eligible for review, as presented in Figure 1 [36-116]. Overall, the included studies were assessed as having low-to-moderate risk when evaluated with the ROBINS-I tool, suggesting that the overall quality of the included papers was high.

**Figure 1.**
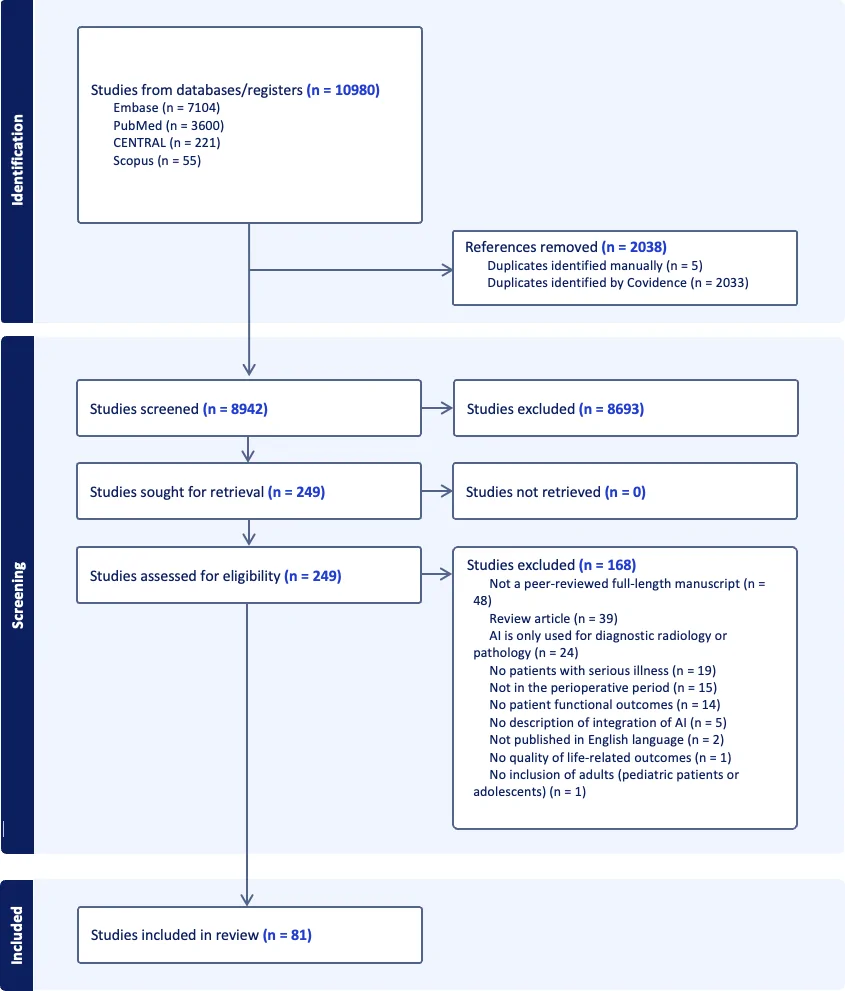
Preferred Reporting Items for Systematic Reviews and Meta-Analysis Protocols (PRISMA) flowchart.

### Characteristics of Sources of Evidence

Across all studies, there was an overall total of 714,253 participants. A majority of the studies were published after 2022, with 59 studies published during or after 2022 and 22 studies published prior to 2022. A majority of the studies were published in China (35 studies), with the United States (9 studies) and South Korea (7 studies) having the next highest numbers of publications, as shown in Figure 2. Of the 81 included studies, 80 (98.8%) focused on patients with malignancy, suggesting a growing interest and importance of AI integration within oncology specifically. The most common forms of cancer in the included papers were colorectal cancer (15 studies), hepatic cancer (11 studies), and head and neck cancer (9 studies). One study focused on patients with liver failure requiring surgery, without a primary malignancy in all patients [109]. A majority of studies used AI for prognostication (73 studies), with 4 using it for risk scores, 3 for treatment recommendations, and 1 using AI for decision support. All studies used ML as their form of AI and included different models, including support vector machine (SVM), least absolute shrinkage and selection operator (LASSO) regression, and extreme gradient boosting (XGBoost). Of the included papers, 75 were retrospective studies, and 6 were prospective cohort studies. A comprehensive overview of relevant study characteristics can be found in Multimedia Appendices 3 and 4.

**Figure 2.**
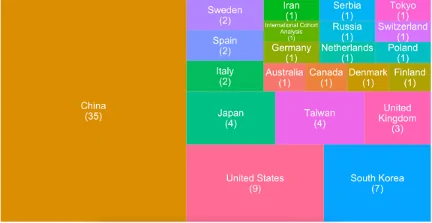
Country representation of included studies by frequency.

### Results of Individual Sources of Evidence and Synthesis of Results

#### AI Equity

##### Consideration of Model Use in Low-Resource Settings (n=17)

Seven study teams addressed the potential for their models to be deployed in low-resource settings. Several study teams explained that model inputs were intentionally selected for their ease of acquisition in standard patient care settings (as opposed to data points that may require costly equipment to obtain or advanced analytical software to calculate) [46,59,84,91]. Two study teams specifically called out the low-cost nature of their model development [84,109], and one referenced their model’s convenience and potential for widespread distribution relative to comparable existing options [103]. One study team originally stated that their ideal model application would be an electronic health record (EHR)–integrated tool, but included a statement about the potential for the creation of a standalone mobile application for use in low-resource settings that may not have robust information technology infrastructure [45]. These discussions reflect an effort to mitigate structural inequities in technological capacity and underscore the importance of context-aware model design for advancing AI equity in diverse care environments.

##### Distribution of an Open-Access Tool (n=6)

Six of the studies included in our sample described the creation of a web-based tool that would allow clinicians outside their organizations to implement the model and generate insights for their own patients [41,61,62,78, 86,91], reinforcing a commitment to equitable access to these emerging technologies.

##### Design Considerations for Non-–AI-Expert Clinicians (n=4)

Usability for frontline clinicians, regardless of their expertise in AI model development, was explicitly discussed in 4 studies. These authors described human-centered design choices, such as clear instructions, intuitive interfaces, and simplified input-output mechanisms to facilitate real-world implementation of these tools and to ease communication between patients and their providers [41,84,116]. One study focused on how their tool could be embedded easily into clinical workflows by creating publicly available software that could be incorporated into an EHR for individualized risk prediction [45]. These features reflect a growing recognition that equitable deployment of AI depends not only on tool accuracy but also on ensuring that all clinicians, regardless of AI fluency, can effectively incorporate these models into patient care.

##### Consideration of the Cost Burden to the Patient Resulting From AI Decision Output (n=1)

One study explicitly addressed the potential downstream cost implications of AI-enabled decision-making for patients: Qiao et al [89] cautioned clinicians to interpret model outputs in a manner that avoids unnecessary or overly burdensome interventions, particularly in cases where the AI-generated risk assessments might influence decisions around costly diagnostic or treatment options. This finding highlights an emerging but underdeveloped area of AI equity: namely, the affordability of care pathways shaped by algorithmic outputs. Without attention to the financial consequences of model-informed decisions, there is a risk of exacerbating disparities among patients already facing economic hardship.

### AI Ethics

#### Lack of Patient Sample Diversity (n=16)

Many studies were conducted at a single site and/or involved a retrospective study design, inherently introducing the potential for selection bias and limiting generalizability. This was frequently noted via a brief acknowledgment in the limitations sections of the papers included in our review. However, 16 study teams discussed concerns about selection bias and lack of generalizability more robustly. Our findings indicate that authors primarily relied on 3 techniques to reflect on concerns about lack of patient sample diversity: calling out the specific methodological issues leading to these worries, explaining specific techniques used to address limitations in diversity, and identifying the need for further exploration beyond the scope of the existing work to further validate their models and thereby address these ethical considerations.

Six study teams mentioned specific considerations driving their concerns regarding a lack of representativeness of the study population, ranging from a lack of geographic and demographic diversity in the sample [43,77,89], limited sample sizes [44], and lack of external validation in additional populations [66], to the overrepresentation of patient data from highly resourced settings (eg, hospitals with infrastructure to uphold NSQIP (National Surgical Quality Improvement Program) reporting requirements [61] or from exclusively tertiary care settings [68]).

Other authors mentioned intentional steps taken to enhance the diversity of their analytic sample, including the intentional inclusion of patients from multiple institutions [41,80], strict study recruitment screening guidelines designed to address concerns about population homogeneity [58], and the intentional selection of a patient population that is currently underrepresented in clinical trials [90]. In the manuscript, 1 study team included an explanation of the technique they used to address class-imbalance problems, leading to improved data quality and representativeness of real-world settings [88]. A handful of other studies mentioned concerns about lack of generalizability more generally but also included a call for future work with more diverse populations, inherently implying the need to address ethical considerations of model dissemination [45,50,53,92].

#### Omission of Socially and Clinically Relevant Features Limiting Model Accuracy (n=5)

Concerns about missing or incomplete data elements were discussed in a small subset of studies, primarily in the context of limitations in model performance. For example, several study teams highlighted the absence of key variables, such as potentially relevant clinical characteristics, smoking status, insurance status, and other socioeconomic factors, which have the potential to meaningfully impact model performance [47,57,74].

One study team mentioned that various methodological options are available to impute missing values, which could help avoid the loss of important information [86]. Relatedly, Kadomatsu et al [60] noted that the inclusion of subjective variables (ie, those requiring clinician judgment) may introduce variability or distortion in the dataset, further complicating model accuracy and reliability.

#### Discussion of Health Disparities (n=2)

Only 2 studies included in our review explicitly discussed racial and ethnic disparities in the context of model development or evaluation, despite the clear relevance of these considerations. Osman et al [86] specifically mentioned differences in mortality rates across racial and ethnic groups that are observed in national trends; they highlighted that their model performed consistently across datasets with differing baseline characteristics, thereby improving faith in the model’s robustness. Verma [101] acknowledged that their model’s output identified differences in risks across racial and ethnic groups and called out the need to both understand the mechanism of action behind this finding and to prevent the model from exacerbating existing disparities.

### AI Safety

#### Removing the AI Black Box for Transparency (n=7)

AI safety included clarity and transparency about how the algorithm used the data to generate output and provided insight into what is sometimes thought of as the “black box.” Certain studies emphasized the importance of sharing how the AI algorithm generated results through what they termed a “white box,” since they felt it provides highly interpretable results [55]. This has been echoed by the AMA’s emphasis on explainability and clinician interpretability. Others have emphasized transparency by publishing their code publicly so that others can easily see how the AI model was trained to produce results, and so that the results can be reproduced in other settings [78]. Other studies have argued that increased insight into how AI created the results was important for transparency and to assist surgeon decision-making [79].

#### AI Considers Additional Factors That May Influence Patient Care (n=6)

Although AI can be a powerful tool, it cannot be used in every setting without clinician input, and the final decision regarding patient care should not be made solely by AI. In addition to clinical factors that affect patient outcomes, there are certain socioeconomic factors that can significantly impact a patient’s care that are not fully accounted for in current iterations of AI [60]. Multiple studies have emphasized that although there is great benefit to incorporating AI into serious illness perioperative care, the provider should still make the final decision regarding the patient’s care [43,65].

#### Physical Safety From Additional Testing

Harnessing AI algorithms may also pose a risk of physical harm to patients, threatening AI safety. Liu et al [77] showed that, although there were benefits to incorporating AI, patients were exposed to high radiation from positron emission tomography (PET) scans and incurred increased costs without significant benefit.

### AI-Enabled Team Augmentation

Despite growing attention toward the consideration of AI as true “team members,” none of the included studies used this lens in the narrative discussion of how the tools were being included in workflows [117]. However, the majority of papers described how the AI tool under consideration could be used to improve perioperative care by prompting a provider, team member, or patient/caregiver to take a tangible action.

There were 2 studies in which the AI model specifically prompted the provider in terms of patient care. Wang et al [104] used their model to provide personalized recommendations and solutions that the physicians could then act on as part of the study. Zhang et al [113] crafted a study design where their AI model assisted in distinguishing gallbladder carcinoma from xanthogranulomatous cholecystitis in the preoperative setting, thus directly impacting surgical decision-making in the perioperative period. Seventy-seven of the other included studies did not explicitly use their AI model to prompt providers, team members, or patients/caregivers, but described how their model could be incorporated in health care settings to prompt others and lead to real-time action contributing positively to patient care in the perioperative setting.

## Discussion

### Summary of Evidence

Our study systematically examined the extent to which scholarly work on AI in perioperative care for patients with serious illness explicitly incorporates discussions of equity, ethics, and safety. Only low-to-moderate risk studies, per the ROBINS-I risk of bias tool, were included in this review. However, the ultimate goal of these applications is to use AI-enabled team augmentation that contributes positively to clinician workflow, improves decision-making speed, and enhances overall patient safety. Although numerous studies have focused on the statistical and methodological validation of AI tools in this setting, our findings reveal a significant gap: key ethical considerations—particularly concerning vulnerable patient populations—remain largely unaddressed [14]. Specifically, we noted that limitations in sample diversity and generalizability, often framed by authors in purely methodological terms, carry profound ethical implications for justice and beneficence. By risking unequal performance across diverse patient subgroups and obscuring systemic disparities through the omission of socially and clinically relevant features, these limitations directly challenge the principles required for fair and trustworthy decision-making in real-world clinical applications. Although this study focused primarily on oncology, many core tenets related to the ethics of integrating AI can be extrapolated to other types of serious illnesses, such as advanced organ failure, frailty, dementia, neurodegenerative diseases, and stroke. Therefore, our analysis underscores the critical need for a paradigm shift, moving the conversation beyond technical statistical performance to demand the integration of rigorous, equity-focused analyses into both the validation and the broader implementation planning of AI tools used in the sensitive domain of perioperative patient care by explicitly including equity, ethics, and safety within the implementation of perioperative AI.

The integration of AI into the perioperative care of seriously ill patients presents unique and critical ethical and safety challenges. As a highly vulnerable population, these patients stand to benefit from AI’s ability to analyze complex data for refined decision-making and personalized care, but they are also at increased risk of the potential for algorithmic bias and data privacy breaches [118]. The application of AI must be carefully considered, distinguishing between a patient-facing chatbot and other leveraged forms of AI that may also improve patient care [119]. Ensuring the safe deployment of AI is paramount and necessitates a multifaceted approach, including clearly defined safety parameters, robust risk mitigation strategies, and integrated workflows with essential human oversight [120,121]. Given the transformative potential of AI to fundamentally reshape perioperative care, ensuring robust ethical standards is paramount to safeguard patient safety and maintain trust in these technologies [122]. Currently, a gold standard for addressing the equity, ethics, and safety of AI in the perioperative period is absent from most research articles, a gap that must be addressed in all future manuscripts to align with emerging guidelines such as the US Department of Health and Human Services’ Trustworthy AI Playbook or the AMA’s 8-Step AI Governance Toolkit [8,10,123]. This review helps define the aspects of AI trustworthiness through the definition mentioned in the *Methods* section and encourages all future papers using AI in the perioperative period to explicitly report these criteria to ensure all AI models are held to a rigorous standard for equity, ethics, and safety.

While no existing studies actively use AI as a direct team member, the opportunity to integrate AI in this capacity remains a burgeoning field. The development of trust within human-AI teams is a complex process, and recent research shows that while smaller human-AI teams are initially seen as less trustworthy than human-only groups, this trust deficit diminishes as the team grows, suggesting that team complexity plays a key role in developing trust [124]. AI-powered systems, including chatbots, large language models (LLMs), and agentic AI, are poised to function as effective members of the health care team, either by partnering with providers to deliver clinical decision support or by engaging with patients to facilitate communication, education, and guidance [6,125]. Although minimal literature exists on integrating AI-enabled team augmentation in health care, and none specifically in the context of surgery, AI has the potential to prompt a team member, whether a clinician or a patient. This capacity to prompt offers significant potential to enhance surgical care for seriously ill patients through improved risk stratification, personalized education, and optimized monitoring. However, its successful implementation hinges on addressing critical concerns related to algorithmic bias, data privacy, and patient trust, while also tackling practical challenges related to integration, overreliance, and ensuring patient comprehension, particularly within this vulnerable population. In addition, trust calibration between humans and AI improves after exposure and iterative feedback, suggesting that increased engagement with AI by health care teams may lead to more AI-enabled team augmentation [126].

Moving beyond traditional machine learning models, generative AI presents both unique opportunities and significant pitfalls in the perioperative period. Generative AI offers the potential to create synthetic patient data for enhanced preoperative risk modeling and simulation, allowing for more personalized surgical planning in complex cases of serious illness [127]. However, this necessitates careful validation to ensure these synthetic datasets accurately reflect real-world patient variability and avoid perpetuating biases [127]. Although generative AI can produce tailored patient education materials and postoperative instructions, improving patient understanding and adherence, there is a risk of generating inaccurate or misleading information, requiring rigorous oversight and quality control to maintain patient safety—a risk not as prominent as with more static ML models [128]. Furthermore, generative AI can streamline administrative tasks, such as generating discharge summaries and patient follow-up plans—a benefit not yet realized in ML—thereby freeing clinicians to focus on direct patient care. Despite these opportunities, critical ethical concerns remain regarding data privacy and the potential for algorithmic bias to impact resource allocation and patient prioritization, underscoring the need for careful and deliberate implementation.

### Limitations

This review should be considered with the following limitations. Our broad definitions, used to capture a comprehensive range of AI applications, may have inadvertently led to the exclusion of relevant studies, as the terminology in this constantly evolving field lacks standardization. There were few studies focusing on the workflow integration of AI, which represents a lack of evidence in this space and demonstrates an opportunity to develop this field of research. Due to feasibility constraints, we were unable to conduct a continuous search, and thus, our findings represent a snapshot of a rapidly developing landscape, potentially missing the most recent publications. A significant limitation in the current literature is the scarcity of data on the practical integration of AI models into clinical workflows and a notable absence of discussion regarding ethics, equity, and safety. Furthermore, our study did not analyze the validity or performance of the AI models themselves, focusing instead on their application and integration. Finally, a significant number of included studies were published in China (35 studies), and additional studies published in languages other than English may have been excluded from this study. This limited geographic and demographic diversity may affect generalizability and inherently create a risk of algorithmic bias. Future research should prioritize the exploration of AI-enabled team augmentation and analyze clinical workflows to better understand where and how AI is being integrated into the perioperative period across different regions of the world and in different health care settings. Since a significant majority of papers were in the realm of oncology, further research on AI integration should focus on populations with other serious illnesses. There should also be longer-term follow-up of AI models to truly understand their impact and safety concerns, along with studies that focus on external validation, prospective implementation, and real-world workflow evaluation so that stronger conclusions can be drawn about clinical utility [129]. It is also crucial to explore the perspectives of patients with serious illnesses and their clinicians regarding the ethical implications of AI in perioperative care, examining their concerns, expectations, and preferred levels of AI integration to ensure that future implementation plans are patient-centered and ethically sound.

### Conclusions

This investigation highlights the considerable potential of AI tools for predictive analytics and other AI tools in surgical outcomes while simultaneously exposing a significant deficit in the explicit reporting of ethical considerations, equity, and safety in current publications in the perioperative period. Among the 81 included papers, 80 of which focused on malignancy, there were significant ethical deficiencies, as very few studies mentioned key factors such as racial or ethnic disparities, sample diversity, or socially relevant features. Achieving the clinical viability of these tools requires moving beyond initial statistical reporting to incorporate rigorous external validation, continuous performance monitoring, and fundamental adherence to principles of trustworthiness and safety. The evidence suggests that unless researchers prioritize equity-focused design and data transparency, the risk of perpetuating structural health disparities within this vulnerable perioperative population remains high. To address these critical issues, it would be beneficial for future research to uphold the gold standard of including validation sets, a practice currently lacking in many published studies, and to demand comprehensive data transparency, including information on patient race and ethnicity. By prioritizing prospective studies and transparent reporting, the research community can build the trust necessary for adoption, aligning AI innovation in perioperative care with the broader HHS goals for improving health outcomes through innovative, fair, safe, and ethical technology.

## Supplementary material

10.2196/89936 Multimedia Appendix 1 Search strategy.

10.2196/89936 Multimedia Appendix 2 Abstraction form and ROBINS-I tool.

10.2196/89936 Multimedia Appendix 3 Included study characteristics.

10.2196/89936 Multimedia Appendix 4 Structured synthesis table of included studies.

## Abbreviations

AMA: American Medical Association
ANN: artificial neural network
CENTRAL: Cochrane Central Register of Controlled Trials
EHR: electronic health record
HHS: Health and Human Services
LASSO: least absolute shrinkage and selection operator
LLM: large language model
ML: machine learning
NLP: natural language processing
PET: positron emission tomography
PICOTS: population, intervention, comparator, outcomes, timing, and setting
PRISMA: Preferred Reporting Items for Systematic Reviews and Meta-Analysis Protocols
PROSPERO: International Prospective Register of Systematic Reviews
ROBINS-I: risk of bias tool in nonrandomized studies of interventions
SVM: support vector machine
WHO: World Health Organization
XGBoost: extreme gradient boosting
