# Supplementary material for: AI Trustworthiness in the Perioperative Period for Patients with Serious Illness: Scoping Narrative Review
Source: JMIR Perioper Med. 2026 Oct 1;9:e89936. doi: 10.2196/89936 (PMC13630345; doi:10.2196/89936)
Supplement: Supplementary file 1 [file periop-v9-e89936-s001.docx]

**Appendix S1. Search Strategy**

**PubMed (07/01/2024) - 3,600 results**

“artificial intelligence”[tw] OR "Artificial Intelligence"[Mesh] OR “machine learning”[tw] OR machine-learning[tw] OR “natural language processing”[tw] OR “NLP”[tw] OR “AI/ML”[tw] OR ChatGPT[tw] OR “Chat GPT”[tw] OR “generative AI”[tw] OR “decision tree"[tw] OR “generative models”[tw] OR “AI model”[tiab:~4] OR “AI models”[tiab:~4] OR “ML model”[tiab:~4] OR “ML models”[tiab:~4]

AND

“perioperat*”[tw] OR “perioperati*”[tw] OR “pre-operative”[tw] OR “preoperative”[tw] OR “postoperative”[tw]

AND

"critical illness"[Mesh] OR "chronic disease" [Mesh] OR "terminal care"[Mesh] OR "critical care"[Mesh] OR "serious illness"[tw] OR terminal[tw] OR chronic[tw] OR advanced[tw] OR severity[tw] OR severe[tw] OR failure*[tw] OR end stage[tw] OR endstage[tw] OR dying[tw] OR “chronic pain”[mesh] OR Intensive Care Units[MeSH] OR intensive care[tw] OR ICU[tw] OR hospice*[tw] OR terminally ill*[tw] OR cancer*[tw] OR neoplas*[tw] OR carcinoma*[tw] OR malignan*[tw] OR tumor*[tw] OR oncolog*[tw] OR sarcoma*[tw] OR adenocarcinoma[tw] OR metasta*[tw] OR neoplasms[Mesh] OR tumour[tw] OR tumours[tw] OR leukemia*[tw] OR lymphoma[tw] OR melanoma[tw] OR blastoma[tw]

**EMBASE (06/11/2024) - 7,104 results**

(“artificial intelligence” OR "Artificial Intelligence" OR “machine learning” OR machine-learning OR “AI/ML” OR ChatGPT OR “Chat GPT” OR “generative AI” OR “decision tree” OR “generative models” OR “AI model” OR “AI models” OR “ML model” OR “ML models”)

AND

(“perioperat*” OR “perioperati*” OR “pre-operative” OR “preoperative” OR “postoperative”)

AND

("critical illness" OR "chronic disease" OR "terminal care" OR "critical care" OR "serious illness" OR terminal OR chronic OR advanced OR severity OR severe OR failure* OR end stage OR endstage OR dying OR “chronic pain” OR Intensive Care Units OR intensive care OR ICU OR hospice* OR terminally ill* OR cancer* OR neoplas* OR carcinoma* OR malignan* OR tumor* OR oncolog* OR sarcoma* OR adenocarcinoma OR metasta* OR neoplasms OR tumour OR tumours OR leukemia* OR lymphoma OR melanoma OR blastoma)

AND

'english':la AND [2010-2024]/py

**Scopus (07/01/2024) - 55 results**

(“artificial intelligence” OR "Artificial Intelligence" OR “machine learning” OR machine-learning OR “AI/ML” OR ChatGPT OR “Chat GPT” OR “generative AI” OR “decision tree” OR “generative models” OR “AI model” OR “AI models” OR “ML model” OR “ML models”)

AND

(“perioperat*” OR “perioperati*” OR “pre-operative” OR “preoperative” OR “postoperative”)

AND

("critical illness" OR "chronic disease" OR "terminal care" OR "critical care" OR "serious illness" OR terminal OR chronic OR advanced OR severity OR severe OR failure* OR end stage OR endstage OR dying OR “chronic pain” OR Intensive Care Units OR intensive care OR ICU OR hospice* OR terminally ill* OR cancer* OR neoplas* OR carcinoma* OR malignan* OR tumor* OR oncolog* OR sarcoma* OR adenocarcinoma OR metasta* OR neoplasms OR tumour OR tumours OR leukemia* OR lymphoma OR melanoma OR blastoma)

**Cochrane Central Register of Controlled Trials (CENTRAL) (07/01/2024) - 221 results**

(“artificial intelligence” OR "Artificial Intelligence" OR “machine learning” OR machine-learning OR “AI/ML” OR ChatGPT OR “Chat GPT” OR “generative AI” OR “decision tree” OR “generative models” OR “AI model” OR “AI models” OR “ML model” OR “ML models”)

AND

(“perioperat*” OR “perioperati*” OR “pre-operative” OR “preoperative” OR “postoperative”)

AND

("critical illness" OR "chronic disease" OR "terminal care" OR "critical care" OR "serious illness" OR terminal OR chronic OR advanced OR severity OR severe OR failure* OR end stage OR endstage OR dying OR “chronic pain” OR Intensive Care Units OR intensive care OR ICU OR hospice* OR terminally ill* OR cancer* OR neoplas* OR carcinoma* OR malignan* OR tumor* OR oncolog* OR sarcoma* OR adenocarcinoma OR metasta* OR neoplasms OR tumour OR tumours OR leukemia* OR lymphoma OR melanoma OR blastoma)
