# Supplementary material for: AI Trustworthiness in the Perioperative Period for Patients with Serious Illness: Scoping Narrative Review
Source: JMIR Perioper Med. 2026 Oct 1;9:e89936. doi: 10.2196/89936 (PMC13630345; doi:10.2196/89936)
Supplement: Supplementary file 2 [file periop-v9-e89936-s002.docx]

**Appendix S2. Abstraction Form**

Abstraction Form A

**Identification:**

**Study Details**

Country:

Last Name of First Author:

Publication date:

**Baseline Characteristics**

| **Characteristics** | **Overall** |
| --- | --- |
| **Gender** | N male (%)  N female (%)  N other (%) |
| **Age**  Mean (SD); Range |  |
| **Race/Ethnicity** | American Indian/Alaska Native: N (%)    Asian: N (%)    Black or African American:  N (%)    Native Hawaiian/Other Pacific Islander: N (%)    Latinx: N (%)    White: N (%) |
| **Category of serious illness(s)** | 1. **Malignancy**    a. Any type of malignant cancer. Benign cancers will not be included 2. **Advanced Organ Failure**    a. Must be irreversible (unless patient gets an organ transplant) and chronic (no acute processes such as infection)    b. Common examples: end stage heart failure, COPD, cirrhosis, end stage renal disease 3. **Frailty + Dementia/Neurodegenerative Disease**    a. Chronic, irreversible, progressive, and deteriorating conditions with no cure    b. Multiple chronic conditions, significant functional impairments, and an increased risk of mortality    c. Common examples: Dementia, Parkinson's Disease 4. **Stroke with Significant Residual Impairment**    a. Major stroke and have experienced significant and persistent neurological deficits |

**Interventions**

Overall Directions: *Some boxes will not be applicable to certain studies, and thus can be filled in with “N/A.”*

| **Question** | **Intervention** |
| --- | --- |
| **What was AI utilized for in the intervention?** | 1. **Prognostication:**    a. AI models are used to help predict the likelihood of future health events, such as postoperative complications, readmission risk, or mortality, by analyzing patient data. 2. **Screening and Triage:**    a. AI models are used to triage patients based on symptom severity and risk factors, helping to prioritize care and optimize resource allocation. 3. **Decision Support:**    a. AI models are used to provide clinicians with evidence-based recommendations and insights during diagnosis and treatment planning. 4. **Treatment Recommendations:**    a. AI models are used to recommend personalized treatment options based on patient characteristics, medical history, and the latest medical literature. 5. **Risk Scores:**    a. AI models are used to develop and refine risk scores for various conditions, to help identify high-risk individuals who may not be fit for surgical intervention. 6. **Medical Chatbot:**    a. AI-powered chatbots can be used to answer patient queries and provide basic medical information. |
| **What category of AI was used?** | 1. **Generative AI:**    a. Generative AI focuses on creating new content, such as text, images, or synthetic patient data. Generative AI learns the underlying patterns and structures of the data they are trained on and then use that knowledge to generate entirely new content that is similar in style and characteristics to the training data.    b. E.g. Chat GPT, Gemini, Claude 2. **Machine Learning (ML):**    a. ML algorithms learn patterns from data without explicit programming, enabling them to make predictions or decisions.    b. E.g. Support vector machine (SVM). Least absolute shrinkage and selection operator (LASSO) regression; Extreme gradient boosting (XGBoost), Random Forest 3. **Natural Language Processing (NLP):**    a. NLP focuses on enabling computers to understand, interpret, and generate human language.    b. E.g. Siri, Alexa 4. **Artificial Neural Network (ANN):**    a. ANN is a computing system inspired by the human brain, composed of interconnected nodes that process information and learn from data to perform tasks like classification, prediction, and pattern recognition.    b. E.g. Sybil, image recognition softwares |
| **What AI interface/tool was used?** | *AI model name* |
| **Was AI equity mentioned in the paper?** | *Yes/ No*  *Definition of AI equity:* The fair and just distribution of the benefits and opportunities afforded by AI technologies across all populations, regardless of race, ethnicity, gender, socioeconomic status, geographic location, or other social determinants of health, while also mitigating any potential harms or biases that could exacerbate existing health disparities.  *Example:* “In addition to reporting our methods and results in this paper, we created a web application accessible to physicians worldwide.” |
| **If equity was mentioned, how?** |  |
| **Was AI ethics mentioned in the paper?** | *Yes/ No*  *Definition of AI ethics:* Ensuring that the development, implementation, and use of AI adhere to the fundamental ethical principles of beneficence, non-maleficence, autonomy, justice, and explainability, while mitigating potential risks, such as biased screening data, to promote equitable and trustworthy healthcare outcomes.  *Example:* “This study used registry data to obtain a more accurate depiction of breast cancer surgery treatment in large populations. Using registry data also minimizes referral bias or bias caused by the practices of a single physician or a single institution.” |
| **If ethics was mentioned, how?** |  |
| **Was AI safety mentioned in the paper?** | *Yes/ No*  *Definition of AI safety:* The development and implementation of measures and practices that ensure AI used in medical settings are reliable, robust, and function as intended, minimizing the risk of harm to patients, clinicians, or the healthcare system.  *Example:* “Recognizing the ‘AI black box,’ we published exactly how our model analyzed the data inputted and created the final results.” |
| **If safety was mentioned, how?** |  |
| **Was AI utilized as a team member?** | *Yes/ No* |
| **If AI was utilized as a team member, how?** |  |
| **Was AI utilized to prompt someone to do something (e.g. patient or clinician)?** | *Yes/ No* |
| **If AI was utilized to prompt someone, how?** |  |

ROBINS-I Tool

**Confounding:** Baseline confounding occurs when one or more prognostic variables (factors that predict the outcome of interest) also predicts the intervention received at baseline.

**Selection of participants in the study:** When exclusion of some eligible participants, or the initial follow-up time of some participants, or some outcome events is related to both intervention and outcome, there will be an association between interventions and outcome even if the effects of the interventions are identical.

**Classification of intervention:** Bias in classification of interventions Bias introduced by either differential or non-differential misclassification of intervention status.

**Deviation from intended interventions:** Bias that arises when there are systematic differences between experimental intervention and comparator groups in the care provided, which represent a deviation from the intended intervention(s).

**Missing data:** Bias that arises when later follow-up is missing for individuals initially included and followed (such as differential loss to follow-up that is affected by prognostic factors); bias due to exclusion of individuals with missing information about intervention status or other variables such as confounders.

**Measurement of outcomes:** Bias introduced by either differential or non-differential errors in measurement of outcome data. Such bias can arise when outcome assessors are aware of intervention status, if different methods are used to assess outcomes in different intervention groups, or if measurement errors are related to intervention status or effects.

**Selection of the reported results:** Selective reporting of results in a way that depends on the findings and prevents the estimate from being included in a meta-analysis (or other synthesis).

Abstraction Form B - In Depth for Generative AI

**Identification:**

**Study Details**

Sponsorship source: *The source of funding for this project.*

Country:

Publication date:

**Author’s contact details**

Author’s name:

Institution:

Email:

Address:

**Methods**

Study Design:

**Population**

Inclusion Criteria: *Write out the study’s inclusion criteria for participants*

Exclusion Criteria: *Write out the study’s exclusion criteria for participants*

Group Differences: *If no differences, type “None;” If study design only includes one group and group differences are not relevant, type “N/A.”*

**Baseline Characteristics**

| **Characteristics** | **Intervention Arm** | **Control** | **Overall** |
| --- | --- | --- | --- |
| **Sample Size** (N) |  |  |  |
| **Gender** | N male (%)  N female (%)  N other (%) | N male (%)  N female (%)  N other (%) | N/A |
| **Age**  Mean (SD); Range |  |  | N/A |
| **Race/Ethnicity** | American Indian/Alaska Native: N (%)    Asian: N (%)    Black or African American:  N (%)    Native Hawaiian/Other Pacific Islander: N (%)    Hispanic or Latino: N (%)    White: N (%) |  | N/A |
| **Type of serious illness(s)** | *What type of serious illness are the participants of the study suffering from (eg. dementia, stroke, cancer, etc…)?* |  |  |
| **Surgery Type/ Surgical Procedure Planned** |  |  |  |
| **Setting** | *Where is the* ***intervention*** *taking place (eg. in-patient/hospital, clinic based/ambulatory, skilled nursing facilities, home, other)?* |  |  |

**Interventions**

Overall Directions: *Some boxes will not be applicable to certain studies, and thus can be filled in with “N/A.”*

| **Question** | **Intervention Arm** | **Control** |
| --- | --- | --- |
| **Arm description** | *Copy paste all the details included in the article* | *Copy paste all the details included in the article* |
| **At what time point (frequency/ timing) was data collected for an AI model?** | *E.g. Pre-operatively, post-operative, throughout perioperative period + what timing (pre-operatively, 3 weeks before surgery)*  *E.g. one time, continuously post-operative, once a week for 4 weeks* |  |
| **At what time point (frequency/ timing) was AI integrated in the perioperative period?** | *E.g. Pre-operatively, post-operative, throughout perioperative period + what timing (pre-operatively, 3 weeks before surgery)*  *E.g. one time, continuously post-operative, once a week for 4 weeks* |  |
| **How was AI integrated/ what did AI do?** |  |  |
| **What implementation framework was used to integrate AI into workflow? If so, which one?** |  |  |
| **What category was AI utilized for (e.g. Screening and triage; Prognostication; Decision support; Treatment recommendations; Risk scores; Clerical support)?** |  |  |
| **Was there training in the use of AI?** | *Of patients or caregivers or care team members* |  |
| **What AI interface/tool was used?** | *E.g. AI model name* |  |
| **What persons/roles utilized AI?** | *Eg. Surgeons, nurses, patients, caregivers* |  |
| **Was AI utilized to prompt someone (physician, healthcare worker, patient) to do something? Will AI be used to prompt others in the future?** |  |  |
| **Was AI equity mentioned in the paper? If so, how?** |  |  |
| **What were the patient quality-related outcomes?** | *List all the patient quality outcomes* | *List all the patient quality outcomes* |
| **Did patient quality-related outcomes improve?** | *If yes, list which outcomes improved and designate if significant improvement.*  *If not, list the major outcomes that were measured and indicate there was no significant improvement.* | *If yes, list which outcomes improved and designate if significant improvement.*  *If not, list the major outcomes that were measured and indicate there was no significant improvement.* |

**Outcomes**

Overall Directions: Each study will likely have different outcomes and different ways they are reporting the outcomes, thus, individual tables will have to be built for each article (some articles might have multiple usable outcomes and will have multiple tables that we will extract).

****This step will be different per article based on the results and will be completed within Covidence itself**
