# Supplementary material for: AI Trustworthiness in the Perioperative Period for Patients with Serious Illness: Scoping Narrative Review
Source: JMIR Perioper Med. 2026 Oct 1;9:e89936. doi: 10.2196/89936 (PMC13630345; doi:10.2196/89936)
Supplement: Supplementary file 3 [file periop-v9-e89936-s003.docx]

**Appendix S3. Included Study Characteristics**

| **Study** | **Patient Characteristics** | **What was AI utilized for in the intervention?** | **What AI interface/tool was used?** | **AI Equity?** | **AI Ethics?** | **AI Safety?** |
| --- | --- | --- | --- | --- | --- | --- |
| Amparore 2024 | 227 patients (Average age: 67.1) with prostate cancer | Prognostication | XGBoost, a gradient boosting machine learning algorithm; Random Forest (ensemble learning algorithm) | No | No | No |
| Cai 2024 | 2044 patients (average age: unknown) with colorectal cancer | Prognostication | Support vector machine (SVM); Least absolute shrinkage and selection  operator (LASSO) regression; Extreme gradient boosting (XGBoost) | No | No | No |
| Chen 2018 | 13399 patients (average age: 59.09) with colorectal cancer | Prognostication | Gradient boosting machine (GBM). GBM is a robust ML method similar to random forest that creates an ensemble of simple decision trees. | No | No | No |
| Choi 2021 | 313 patients (average age: 64.76) with laryngeal and/or hypopharyngeal cancer | Prognostication | Random forest (RF), neural network (NN) algorithms, gradient boosting | No | No | No |
| Constantino 2024 | 193 patients (average age: 63.1) with oral and oropharyngeal cancer | Prognostication | Six 2-class supervised ML decision models: Decision tree models, flexible discriminant analysis, penalized discriminant analysis, random forest models, support vector machines, and extreme gradient boosting | No | No | No |
| Cui 2024 | 220 patients (average age: 60) with metastatic spinal cancer, Thyroid cancer 5 (2.3)  Prostate cancer 31  Breast cancer 20 (9.1)  Renal cancer 43 (19.5)  Lung cancer 58 (26.4)  Hepatocellular carcinoma 13 (5.9)  Gastrointestinal system cancer 12 (5.5)  Urogenital cancer 8 (3.6)  Others 30 (13.6) | Risk Scores | Extreme gradient boosting machine (eXGBM), Support vector machine (SVM), Random forest (RF), Neural network  (NN), and Decision tree (DT) | Yes | Yes | Yes |
| Du 2024 | 670 patients (average age: 50.4) with breast cancer | Prognostication | LASSO Regression, Support Vector Machines, Stochastic Gradient Descent, K-nearest Neighbor, Decision Trees, Random Forests, Extra Trees, Extreme Gradient Boosting, Light Gradient Boosting | No | No | No |
| Fourman 2023 | 104 patients (average age: 56.7) with breast cancer | Prognostication | 2-step unsupervised cluster analysis | No | Yes | Yes |
| Fu 2023 | 119 patients (average age: 60.37) with lung cancer metastasized to spine | Prognostication | Least absolute shrinkage and selection operator (LASSO) regression analysis | No | Yes | No |
| Ganguli 2022 (Machine learning methods to predict presence of residual cancer following hysterectomy.) | 3656 patients (average age: unknown) with patients who underwent hysterectomy for known malignancy | Prognostication | Random Forest, eXtreme Gradient Boosting (XGBoost), Logistic Regression (LR), Support Vector Machine (SVM), and K-Nearest Neighbor  (KNN) algorithms | Yes | Yes | No |
| Ganguli 2022 (Machine learning models to prognose 30-Day Mortality in Postoperative Disseminated Cancer Patients.) | 167,474 patients (average age: unknown) with variable cancer cancer types, undisclosed percentages | Prognostication | Extreme Gradient Boosting, Random Forest, and Logistic Regression machine learning models | Yes | No | No |
| Ghaith 2023 | 9418 patients (average age: 55.8) with supratentorial high-grade gliomas | Prognostication | XGBoost, k-nearest neighbors (KNN), decision tree, naive Bayes, and random forest models | No | Yes | No |
| Guo 2020 | 392 patients (average age: unknown) with pancreatic ductal adenocarcinoma | Prognostication | Nomogram and decision tree models | No | No | No |
| Han 2020 | 1769 patients (average age: 67.7) with various periampullary tumors | Prognostication | Random forest (RF) and Neural network (NN) | No | No | No |
| He 2024 | 3010 patients (average age: 56.15) with hepatocellular carcinoma | Prognostication | Machine learning–based nomogram mode | No | Yes | No |
| Hong 2022 | 91 patients (average age: 54.1) with primary liver cancer | Prognostication | Support Vector Machines (SVM) and Random Forest | No | No | No |
| Huang 2022 (Development and validation of a combined nomogram model based on deep learning contrast-enhanced ultrasound and clinical factors to predict preoperative aggressiveness in pancreatic neuroendocrine neoplasms) | 104 patients (average age: 47.69) with pancreatic neuroendocrine neoplasms | Prognostication | SE-ResNeXt-50 CNN | No | No | No |
| Huang 2022 (Development and Validation of a Machine Learning Prognostic Model for Hepatocellular Carcinoma Recurrence After Surgical Resection.) | 7919 patients (average age: unknown) with hepatocellular carcinoma | Prognostication | Deep Learning-based Survival Model (DeepSurv), Extreme Gradient Boosting (XGBoost), and Random survival forest (RSF) | No | Yes | No |
| Ishankulov 2022 | 26 patients (average age: unknown) with brain gliomas | Prognostication | A random forest of decision trees, logistic regression, support vector machine with different types of the kernel (linear, radial, and polynomial) | No | No | No |
| Ishizaki 2023 | 259 patients (median age: 66) with colon cancer | Treatment Recommendations | Prediction One software with five-fold cross-validation | No | No | Yes |
| Ivanics 2022 | 1013 patients (median age: 59) with hepatocellular carcinoma | Prognostication | CoxNet (regularized Cox regression), survival random forest, survival support vector machine, and DeepSurv | No | No | No |
| Jeon 2023 | 961 patients (average age: unknown) with rectal cancer | Prognostication | Support vector machine (SVM), random forest (RF), and Extreme gradient boosting (XGBoost) | No | Yes | No |
| Jin 2023 | 1252 patients (average age: 59.8) with lung cancer | Prognostication | Concordance index (Cindex), receiver operating characteristic (ROC) curve, calibration curve, Hosmer Lemeshow goodness-of-fit test and decision curve analysis (DCA) | No | Yes | No |
| Jung 2023 | 961 patients (average age: 62.35) with upper GI cancer | Prognostication | classic Cox proportional hazards model; non-linear Cox proportional hazards model (also called DeepSurv); the Gompertz model; several random survival forest models  were analyzed including the classic random survival forest  (RSF) | Yes | No | No |
| Kadomatsu 2024 | 1111 patients (average age: 68.9) with lung cancer | Prognostication | Elastic Net | No | Yes | Yes |
| Karabacak 2023 | 3073 patients (average age: unknown) with spinal tumors | Prognostication | XGBoost, LightGBM, CatBoost, and random forest | Yes | Yes | Yes |
| Karhade 2019 | 1790 patients (average age: unknown) with malignant neoplasm of bone, meninges, or spinal cord | Prognostication | Random Forest algorithms | Yes | No | Yes |
| Kim 2019 | 33 patients (average age: 70.39) with epithelial ovarian cancer | Prognostication | Recurrent neural network long short-term memory  (RNN-LSTM) | No | No | No |
| Kinoshita 2023 | 1049 patients (average age: unknown) with non-small cell lung cancer | Prognostication | eXtreme Gradient Boosting (XGBoost) | No | No | No |
| Klén 2019 | 1099 patients (average age: 68.6) with bladder cancer | Prognostication | Logistic regression with lasso regularization | No | No | Yes |
| Kuo 2018 | 1854 patients (average age: 54) with head and neck cancer | Prognostication | Feed-forward ANN and the LR models | No | Yes | No |
| Kuwayama 2023 | 1687 patients (average age: 67) with gastric cancer | Prognostication | logistic regression (LR), random forest (RF), gradient boosting (GB) and deep neural network (DNN) | No | No | No |
| Laios 2022 (Stratification of Length of Stay Prediction following Surgical Cytoreduction in Advanced High-Grade Serous Ovarian Cancer Patients Using Artificial Intelligence; the Leeds L-AI-OS Score.) | 285 patients (average age: 64) with advanced high-grade serous ovarian cancer | Prognostication | LR methods; support vector machine (SVM) and artificial neural networks (ANN) in backpropagation (BPN) configuration | No | No | No |
| Laios 2022 (Machine learning-based risk prediction of critical care unit admission for advanced stage high grade serous ovarian cancer patients undergoing cytoreductive surgery: The Leeds-Natal score) | 291 patients (average age: 64.2) with advanced high-grade serous ovarian cancer | Risk Scores | non-linear distance models using neural networks (K-Nearest Neighbors (KNN), ANN in Backpropagation Neural Network (BPN) mode, and the models using linear and quadratic discriminant methods (LDA and QDA)). | No | Yes | No |
| Lee 2021 (Evolutionary Learning-Derived Clinical-Radiomic Models for Predicting Early Recurrence of Hepatocellular Carcinoma after Resection.) | 517 patients (average age: 61.7) with hepatocellular carcinoma | Prognostication | Genetic algorithm for predicting recurrence after surgery of liver cancer (GARSL) | No | No | No |
| Lee 2021 (Usefulness of artificial intelligence for predicting recurrence following surgery for pancreatic cancer: Retrospective cohort study) | 4846 patients (median age: 65) with pancreatic cancer | Prognostication | The random forest and the Cox proportional hazards model (the Cox model) | No | No | No |
| Lee 2022 | 282 patients (average age: 63.38) with pancreatic cancer | Prognostication | Artificial neural network, logistic regression, random forest, gradient boosting, and support vector machine | No | No | No |
| Li 2022 | 211 patients (median age: 55) with peritoneal sarcomatosis in retroperitoneal sarcoma | Prognostication | the random survival forest (RSF) and the least absolute shrinkage and selection operator (LASSO) model | No | Yes | No |
| Li 2024 | 403 patients (average age: unknown) with parotid gland tumors | Prognostication | Logistic Regression (Logit), Random Forest (RF), XGBoost (XGB), Artificial Neural Network (ANN), and Support Vector Machine (SVM). | No | No | No |
| Lin 2022 | 23,907 patients (median age: 71) with colorectal cancer | Prognostication | least absolute shrinkage and selection  operator (LASSO) logistic regression, gradient boosting machines, adaboost, random forest, K nearest neighbor, multilayer perceptrons and decision trees | No | No | Yes |
| Lin 2024 | 1087 patients (median age: unknown) with gynecological malignant tumor patients | Prognostication | Decision tree (DT) model and logistic regression (LR) model | No | No | No |
| Liu 2023 (A Nomogram Based on Nutrition-Related Indicators and Computed Tomography Imaging Features for Predicting Preoperative Lymph Node Metastasis in Curatively Resected Esophagogastric Junction Adenocarcinoma) | 638 patients (average age: 62.72) with esophagogastric junction adenocarcinoma | Prognostication | The least absolute shrinkage and selection operator (Lasso) method, | No | No | Yes |
| Liu 2023 (Prediction model for hepatocellular carcinoma recurrence after hepatectomy: Machine learning-based development and interpretation study.) | 315 patients (average age: 50.09) with hepatocellular carcinoma | Prognostication | Multilayer perceptron (MLP), adaptive boosting (AB), bootstrapped aggregating (BAG), logistic regression (LR), gradient boosting machine (GBM), and extreme gradient boost (XGB) | Yes | Yes | Yes |
| Lopez-Lopez 2024 | 585 patients (average age: unknown) with hepatocellular carcinoma and liver metastasis | Prognostication | KNN, Multi-layer Perceptron (MLP), Random Forest (RF), Adaboost, Linear Discriminant Analysis (LDA), Support Vector Regression SVR), ElasticNet, and Linear Regressor | No | No | Yes |
| Lou 2020 | 1140 patients (average age: 52.3) with breast cancer | Prognostication | Artificial Neural Networks (ANN) Model; K-Nearest Neighbor (KNN) Model; Support Vector Machine (SVM) Model; Naïve Bayesian Classifier (NBC) Model; Cox Proportional-Hazards Regression (COX) Model | No | Yes | No |
| Ma 2023 | 279 patients (average age: unknown) with primary hepatic carcinoma | Prognostication | Least absolute shrinkage and selection operator (LASSO) regression | No | No | No |
| Mai 2020 | 353 patients (average age: 48) with hepatocellular carcinoma | Prognostication | Logistic regression (LR) and artificial neural network (ANN) | No | No | No |
| Masum 2022 | 4336 patients (average age: 70.24) with colorectal cancer | Risk Scores | LASSO, EN, K-Nearest Neighbor, CART, SVR, AB, Gradient Boosting Machine, Random Forest, Extra Trees, Support Vector Machine, Multilayer Perceptron, Bidirectonal Long Short-Term Memory | No | No | No |
| Mazaki 2021 | 787 patients (median age: 70) with colon cancer | Prognostication | "Prediction One Machine Learning Software:" Gradient boosting tree and neural netowrk models, as well as ensemble model of both trained models | Yes | No | Yes |
| Obrzut 2019 | 102 patients (average age: 48) with cervical cancer | Prognostication | Probabalistic Neural Networks (PNN) | No | No | No |
| Osman 2022 | 365,888 patients (average age: unknown) with colorectal cancer | Prognostication | Decision Tree, Random Forest, Light Gradient Boosting Machine | Yes | Yes | No |
| Paro 2022 | 1344 patients (median age: 61) with colorectal liver metastases | Prognostication | Only described in a cited paper [ref 18]  Link: https://onlinelibrary-wiley-com.stanford.idm.oclc.org/doi/10.1002/jso.25450 | No | No | No |
| Pera 2022 | 3182 patients (average age: 70.34) with gastric cancer | Prognostication | Cross Validated Elastic regularized logistic regression (cv-Enet); boosting linear regression (glmboost), random forest, and an ensemble model | No | Yes | No |
| Qiao 2024 | 2396 patients (average age: 44.99) with glioma | Prognostication | LASSO to screen and prioritize variables; Cox proportional hazard models; nomograms  ^ need to revisit this list; not sure I'm 100% understanding the analysis steps with this one | Yes | Yes | No |
| Qin 2023 | 1191 patients (median age: 63) with colorectal cancer | Prognostication | random forest, extreme gradient boosting (XGBoost), weighted support vector machine (SVM), multilayer perception (MLP) network, and long short-term memory (LSTM) network, alongside logistic regression | No | Yes | No |
| Sammour 2017 | 402 patients (median age: 73) with colon cancer | Prognostication | IBM Watson Analytics | Yes | Yes | Yes |
| Shahriarirad 2024 | 1832 patients (average age: 50.9) with breast cancer | Prognostication | This study proposed TabNet, an end-to-end deep learning model, to predict SLN involvement in breast cancer patients33. | No | Yes | No |
| Shao 2021 | 1660 patients (average age: 59.08) with gastric adenocarcinoma | Prognostication | logistic regression (LR), random forest (RF), support vector machine (SVM), and XGBoost | No | No | No |
| Shen 2024 | 860 patients (average age: 59.1) with colorectal cancer | Prognostication | LASSO Regression | No | No | No |
| Shi 2012 | 403 patients (average age: 52.21) with breast cancer | Prognostication | Artificial neural network (ANN): specifically, multilayer perceptron (MLP) network | No | No | No |
| Shi 2024 | 200 patients (average age: 60.99) with metastatic spinal tumors | Prognostication | XGBoosting Machine  Logistic Regression  K-Nearest Neighbor  Support Vector Machine  Decision Tree  Random Forest | No | No | No |
| Spelt 2013 | 241 patients (average age: 66) with colorectal cancer with liver mets | Prognostication | Artifical Neural Network-Effective time dependent hazard ratios (HR) for the risk factors; Harrell's C-Index to test performance | No | No | No |
| Sun 2023 | 1179 patients (average age: 51.56) with hepatocellular carcinoma | Prognostication | Adaboost | No | No | No |
| vandeBeld 2024* | 417 patients (average age: 65.1) with esophageal cancer | Prognostication | ResNet18 architecture; long short term memory (LSTM) units; Multimodal Deep Learning Models; Logistic Regression | No | No | No |
| vanNiftrikCHB 2019* | 668 patients (average age: 56) with cranial tumor | Prognostication | Random Forest, Neural Network, Support Vector, Machine and Gradient Boosting | No | No | No |
| Verma 2023* | 12,281 patients (average age: 65.1) with pancreatic cancer | Prognostication | eXtreme Gradient Boosting (XGBoost) | No | Yes | No |
| Vesovic 2023* | 79 patients (average age: 60.24) with lung cancer | Prognostication | Regression metamodel | No | No | No |
| Wang 2023 (Combined application of inflammation-related biomarkers to predict postoperative complications of rectal cancer patients: a retrospective study by machine learning analysis.)* | 493 patients (average age: 66) with rectal cancer | Prognostication | Logistic regression, vector machines, regression trees, random forest plot | Yes | No | No |
| Wang 2023 (Data-Driven Assisted Decision Making for Surgical Procedure of Hepatocellular Carcinoma Resection and Prognostic Prediction: Development and Validation of Machine Learning Models) | 361 patients (average age: 58.8) with hepatocellular carcinoma | Decision Support | Deep neural networks (DNNs), Deep Model of Survival Prediction, Deep Model of Recurrence Prediction | No | No | No |
| Wen 2021 | 5220 patients (average age: 59.62) with rectal cancer | Prognostication | Random forest | No | No | No |
| Wu 2022 | 370 patients (average age: 49.89) with breast cancer | Prognostication | a naïve Bayes model, a k-nearest neighbor (KNN) model, a support vector machine (SVM) model, a logistic regression model, and a multilayer perceptron (MLP) model | No | No | No |
| Xu 2023 | 575 patients (median age: 50) with breast cancer | Prognostication | Logistic regression (LR), eXtreme Gradient Boosting (XGBoost), k-Nearest Neighbor (KNN), and Support Vector Machine (SVM) | No | No | No |
| Ying 2021* | 292 patients (median age: 74) with bladder cancer | Prognostication | Convolutional Neural Network | No | No | No |
| Zaver 2023* | 712 patients (median age: unknown) with liver failure (requiring transplantation) | Risk Scores | Convolutional neural network-based algorithm | Yes | No | No |
| Zeng 2023 | 1841 patients (average age: unknown) with breast cancer | Prognostication | Three AI algorithms: SVM,  XGBoost, and LSTM | No | No | No |
| Zeng 2024 | 11,076 patients (average age: unknown) with gastric cancer | Prognostication | DeepSurv Neural Network | No | No | No |
| Zhang 2023 | 1901 patients (average age: 55) with medullary thyroid cancer | Prognostication | Random forest classifier (RFC), gradient boosting decision tree (GBDT), logistic regression (LR), and support vector machine (SVM) | No | No | No |
| Zhang 2024 (Development and comparison of machine-learning models for predicting prolonged postoperative length of stay in lung cancer patients following video-assisted thoracoscopic surgery.) | 6767 patients (average age: 56.2) with lung cancer | Prognostication | XGBoost, random forest, neural networks | Yes | No | No |
| Zhang 2024 (Deep learning nomogram for preoperative distinction between Xanthogranulomatous cholecystitis and gallbladder carcinoma: A novel approach for surgical decision) | 371 patients (average age: demographics only include one cohort) with gallbladder carcinoma | Decision Support | FConv-ResNet18 (Fourier transform-based ResNet model) | No | No | No |
| Zhao 2023 | 243 patients (average age: 44.69) with thyroid cancer | Prognostication | LASSO/nomogram prediction model | No | No | No |
| Zheng 2023 | 2875 patients (median age: 61) with gastric cancer | Treatment Recommendations | LASSO Cox Regression | Yes | No | No |
