# Supplementary material for: AI Trustworthiness in the Perioperative Period for Patients with Serious Illness: Scoping Narrative Review
Source: JMIR Perioper Med. 2026 Oct 1;9:e89936. doi: 10.2196/89936 (PMC13630345; doi:10.2196/89936)
Supplement: Supplementary file 4 [file periop-v9-e89936-s004.docx]

**Appendix S4. Structured Synthesis Table of Included Studies**

| **Study** | **Study Type** | **Disease Category** | **What AI interface/tool was used?** | **Sensitivity** | **Specificity** | **ROC** | **Validation Set** |
| --- | --- | --- | --- | --- | --- | --- | --- |
| Amparore 2024 | Prospective Cohort Study | Prostate Cancer | XGBoost, a gradient boosting machine learning algorithm; Random Forest (ensemble learning algorithm) | No | No | AUC/ AUROC reported | No |
| Cai 2024 | Retrospective Study | Colorectal Cancer | Support vector machine (SVM); Least absolute shrinkage and selection  operator (LASSO) regression; Extreme gradient boosting (XGBoost) | Yes | Yes | AUC/ AUROC reported | Yes |
| Chen 2018 | Retrospective Study | Colorectal Cancer | Gradient boosting machine (GBM). GBM is a robust ML method similar to random forest that creates an ensemble of simple decision trees. | No | No | AUC/ AUROC reported | Yes |
| Choi 2021 | Retrospective Study | Laryngeal and/or Hypopharyngeal cancer | Random forest (RF), neural network (NN) algorithms, gradient boosting | No | No | AUC/ AUROC reported | Yes |
| Constantino 2024 | Retrospective Study | Oral and Oropharygeal Cancer | Six 2-class supervised ML decision models: Decision tree models, flexible discriminant analysis, penalized discriminant analysis, random forest models, support vector machines, and extreme gradient boosting | Yes | Yes | AUC/ AUROC reported | Yes |
| Cui 2024 | Retrospective Study | Malignancy | Extreme gradient boosting machine (eXGBM), Support vector machine (SVM), Random forest (RF), Neural network  (NN), and Decision tree (DT) | Yes | Yes | AUC/ AUROC reported | Yes |
| Du 2024 | Retrospective Study | Breast Cancer | LASSO Regression, Support Vector Machines, Stochastic Gradient Descent, K-nearest Neighbor, Decision Trees, Random Forests, Extra Trees, Extreme Gradient Boosting, Light Gradient Boosting | Yes | Yes | AUC/ AUROC reported | Yes |
| Fourman 2023 | Retrospective Study | Breast Cancer | 2-step unsupervised cluster analysis | No | No | No | No |
| Fu 2023 | Prospective Cohort Study | Lung Cancer | Least absolute shrinkage and selection operator (LASSO) regression analysis | Yes | Yes | AUC/ AUROC reported | Yes |
| Ganguli 2022 (Machine learning methods to predict presence of residual cancer following hysterectomy.) | Retrospective Study | Malignancy | Random Forest, eXtreme Gradient Boosting (XGBoost), Logistic Regression (LR), Support Vector Machine (SVM), and K-Nearest Neighbor  (KNN) algorithms | No | No | AUC/ AUROC reported | Yes |
| Ganguli 2022 (Machine learning models to prognose 30-Day Mortality in Postoperative Disseminated Cancer Patients.) | Retrospective Study | Malignancy | Extreme Gradient Boosting, Random Forest, and Logistic Regression machine learning models | No | No | AUC/ AUROC reported | Yes |
| Ghaith 2023 | Retrospective Study | Supratentorial High-Grade Gliomas | XGBoost, k-nearest neighbors (KNN), decision tree, naive Bayes, and random forest models | Yes | Yes | AUC/ AUROC reported | Yes |
| Guo 2020 | Retrospective Study | Pancreatic Ductal Adenocarcinoma | Nomogram and decision tree models | Yes | Yes | No | Yes |
| Han 2020 | Retrospective Study | Periampullary Tumors | Random forest (RF) and Neural network (NN) | No | No | AUC/ AUROC reported | Yes |
| He 2024 | Retrospective Study | Hepatocellular Carcinoma | Machine learning–based nomogram mode | Yes | Yes | AUC/ AUROC reported | Yes |
| Hong 2022 | Retrospective Study | Primary Liver Cancer | Support Vector Machines (SVM) and Random Forest | Yes | Yes | AUC/ AUROC reported | Yes |
| Huang 2022 (Development and validation of a combined nomogram model based on deep learning contrast-enhanced ultrasound and clinical factors to predict preoperative aggressiveness in pancreatic neuroendocrine neoplasms) | Retrospective Study | Pancreatic Neuroendocrine Neoplasms | SE-ResNeXt-50 CNN | Yes | Yes | AUC/ AUROC reported | Yes |
| Huang 2022 (Development and Validation of a Machine Learning Prognostic Model for Hepatocellular Carcinoma Recurrence After Surgical Resection.) | Retrospective Study | Hepatocellular Carcinoma | Deep Learning-based Survival Model (DeepSurv), Extreme Gradient Boosting (XGBoost), and Random survival forest (RSF) | No | No | No | Yes |
| Ishankulov 2022 | Retrospective Study | Brain Gliomas | A random forest of decision trees, logistic regression, support vector machine with different types of the kernel (linear, radial, and polynomial) | Yes | Yes | AUC/ AUROC reported | Yes |
| Ishizaki 2023 | Retrospective Study | Colon Cancer | Prediction One software with five-fold cross-validation | No | No | AUC/ AUROC reported | Yes |
| Ivanics 2022 | Retrospective Study | Hepatocellular Carcinoma | CoxNet (regularized Cox regression), survival random forest, survival support vector machine, and DeepSurv | No | No | No | No |
| Jeon 2023 | Retrospective Study | Rectal Cancer | Support vector machine (SVM), random forest (RF), and Extreme gradient boosting (XGBoost) | Yes | Yes | AUC/ AUROC reported | Yes |
| Jin 2023 | Retrospective Study | Lung Cancer | Concordance index (Cindex), receiver operating characteristic (ROC) curve, calibration curve, Hosmer Lemeshow goodness-of-fit test and decision curve analysis (DCA) | No | No | AUC/ AUROC reported | Yes |
| Jung 2023 | Prospective Cohort Study | Upper GI Cancer | classic Cox proportional hazards model; non-linear Cox proportional hazards model (also called DeepSurv); the Gompertz model; several random survival forest models  were analyzed including the classic random survival forest  (RSF) | No | No | AUC/ AUROC reported | Yes |
| Kadomatsu 2024 | Retrospective Study | Lung Cancer | Elastic Net | No | No | AUC/ AUROC reported | Yes |
| Karabacak 2023 | Retrospective Study | Spinal Tumors | XGBoost, LightGBM, CatBoost, and random forest | Yes | Yes | AUC/ AUROC reported | Yes |
| Karhade 2019 | Retrospective Study | Malignant Neoplasm of Bone, Meninges, or Spinal Cord | Random Forest algorithms | No | No | AUC/ AUROC reported | Yes |
| Kim 2019 | Retrospective Study | Epithelial Ovarian Cancer | Recurrent neural network long short-term memory  (RNN-LSTM) | Yes | Yes | AUC/ AUROC reported | Yes |
| Kinoshita 2023 | Retrospective Study | Non-small Cell Lung Cancer | eXtreme Gradient Boosting (XGBoost) | No | No | AUC/ AUROC reported | No |
| Klén 2019 | Retrospective Study | Bladder Cancer | Logistic regression with lasso regularization | No | No | AUC/ AUROC reported | Yes |
| Kuo 2018 | Retrospective Study | Head and Neck Cancer | Feed-forward ANN and the LR models | Yes | Yes | AUC/ AUROC reported | Yes |
| Kuwayama 2023 | Retrospective Study | Gastric Cancer | logistic regression (LR), random forest (RF), gradient boosting (GB) and deep neural network (DNN) | No | No | AUC/ AUROC reported | Yes |
| Laios 2022 (Stratification of Length of Stay Prediction following Surgical Cytoreduction in Advanced High-Grade Serous Ovarian Cancer Patients Using Artificial Intelligence; the Leeds L-AI-OS Score.) | Retrospective Study | Ovarian Cancer | LR methods; support vector machine (SVM) and artificial neural networks (ANN) in backpropagation (BPN) configuration | Yes | Yes | No | Yes |
| Laios 2022 (Machine learning-based risk prediction of critical care unit admission for advanced stage high grade serous ovarian cancer patients undergoing cytoreductive surgery: The Leeds-Natal score) | Retrospective Study | Ovarian Cancer | non-linear distance models using neural networks (K-Nearest Neighbors (KNN), ANN in Backpropagation Neural Network (BPN) mode, and the models using linear and quadratic discriminant methods (LDA and QDA)). | Yes | Yes | AUC/ AUROC reported | Yes |
| Lee 2021 (Evolutionary Learning-Derived Clinical-Radiomic Models for Predicting Early Recurrence of Hepatocellular Carcinoma after Resection.) | Retrospective Study | Hepatocellular Carcinoma | Genetic algorithm for predicting recurrence after surgery of liver cancer (GARSL) | No | No | AUC/ AUROC reported | Yes |
| Lee 2021 (Usefulness of artificial intelligence for predicting recurrence following surgery for pancreatic cancer: Retrospective cohort study) | Retrospective Study | Pancreatic Cancer | The random forest and the Cox proportional hazards model (the Cox model) | No | No | No | Yes |
| Lee 2022 | Retrospective Study | Pancreatic Cancer | Artificial neural network, logistic regression, random forest, gradient boosting, and support vector machine | Yes | Yes | AUC/ AUROC reported | Yes |
| Li 2022 | Retrospective Study | Peritoneal Sarcomatosis in Retroperitoneal Sarcoma | the random survival forest (RSF) and the least absolute shrinkage and selection operator (LASSO) model | Yes | Yes | AUC/ AUROC reported | Yes |
| Li 2024 | Retrospective Study | Parotid Gland Tumors | Logistic Regression (Logit), Random Forest (RF), XGBoost (XGB), Artificial Neural Network (ANN), and Support Vector Machine (SVM). | Yes | Yes | AUC/ AUROC reported | Yes |
| Lin 2022 | Retrospective Study | Colorectal Cancer | least absolute shrinkage and selection  operator (LASSO) logistic regression, gradient boosting machines, adaboost, random forest, K nearest neighbor, multilayer perceptrons and decision trees | Yes | Yes | AUC/ AUROC reported | Yes |
| Lin 2024 | Retrospective Study | Gynecological Malignant Tumors | Decision tree (DT) model and logistic regression (LR) model | Yes | Yes | AUC/ AUROC reported | Yes |
| Liu 2023 (A Nomogram Based on Nutrition-Related Indicators and Computed Tomography Imaging Features for Predicting Preoperative Lymph Node Metastasis in Curatively Resected Esophagogastric Junction Adenocarcinoma) | Retrospective Study | Esophagogastric Junction Adenocarcinoma | The least absolute shrinkage and selection operator (Lasso) method, | Yes | Yes | AUC/ AUROC reported | Yes |
| Liu 2023 (Prediction model for hepatocellular carcinoma recurrence after hepatectomy: Machine learning-based development and interpretation study.) | Retrospective Study | Hepatocellular Carcinoma | Multilayer perceptron (MLP), adaptive boosting (AB), bootstrapped aggregating (BAG), logistic regression (LR), gradient boosting machine (GBM), and extreme gradient boost (XGB) | Yes | Yes | AUC/ AUROC reported | Yes |
| Lopez-Lopez 2024 | Retrospective Study | Hepatocellular Carcinoma and Liver Metastasis | KNN, Multi-layer Perceptron (MLP), Random Forest (RF), Adaboost, Linear Discriminant Analysis (LDA), Support Vector Regression SVR), ElasticNet, and Linear Regressor | No | No | No | Yes |
| Lou 2020 | Prospective Cohort Study | Breast Cancer | Artificial Neural Networks (ANN) Model; K-Nearest Neighbor (KNN) Model; Support Vector Machine (SVM) Model; Naïve Bayesian Classifier (NBC) Model; Cox Proportional-Hazards Regression (COX) Model | Yes | Yes | AUC/ AUROC reported | Yes |
| Ma 2023 | Retrospective Study | Primary Hepatic Carcinoma | Least absolute shrinkage and selection operator (LASSO) regression | No | No | AUC/ AUROC reported | Yes |
| Mai 2020 | Retrospective Study | Hepatocellular Carcinoma | Logistic regression (LR) and artificial neural network (ANN) | No | No | AUC/ AUROC reported | Yes |
| Masum 2022 | Retrospective Study | Colorectal Cancer | LASSO, EN, K-Nearest Neighbor, CART, SVR, AB, Gradient Boosting Machine, Random Forest, Extra Trees, Support Vector Machine, Multilayer Perceptron, Bidirectonal Long Short-Term Memory | Yes | Yes | ROC plot but no AUC value | Yes |
| Mazaki 2021 | Retrospective Study | Colon Cancer | "Prediction One Machine Learning Software:" Gradient boosting tree and neural netowrk models, as well as ensemble model of both trained models | No | No | AUC/ AUROC reported | Yes |
| Obrzut 2019 | Retrospective Study | Cervical Cancer | Probabalistic Neural Networks (PNN) | Yes | Yes | AUC/ AUROC reported | Yes |
| Osman 2022 | Retrospective Study | Colorectal Cancer | Decision Tree, Random Forest, Light Gradient Boosting Machine | Yes | Yes | AUC/ AUROC reported | Yes |
| Paro 2022 | Retrospective Study | Colorectal Liver Metastases | Only described in a cited paper [ref 18]  Link: https://onlinelibrary-wiley-com.stanford.idm.oclc.org/doi/10.1002/jso.25450 | Yes | Yes | No | Yes |
| Pera 2022 | Retrospective Study | Gastric Cancer | Cross Validated Elastic regularized logistic regression (cv-Enet); boosting linear regression (glmboost), random forest, and an ensemble model | No | No | No | Yes |
| Qiao 2024 | Retrospective Study | Glioma | LASSO to screen and prioritize variables; Cox proportional hazard models; nomograms  ^ need to revisit this list; not sure I'm 100% understanding the analysis steps with this one | Yes | Yes | AUC/ AUROC reported | Yes |
| Qin 2023 | Retrospective Study | Colorectal Cancer | random forest, extreme gradient boosting (XGBoost), weighted support vector machine (SVM), multilayer perception (MLP) network, and long short-term memory (LSTM) network, alongside logistic regression | Yes | Yes | AUC/ AUROC reported | Yes |
| Sammour 2017 | Retrospective Study | Colon Cancer | IBM Watson Analytics | Yes | Yes | AUC/ AUROC reported | Yes |
| Shahriarirad 2024 | Retrospective Study | Breast Cancer | This study proposed TabNet, an end-to-end deep learning model, to predict SLN involvement in breast cancer patients33. | Yes | Yes | AUC/ AUROC reported | Yes |
| Shao 2021 | Retrospective Study | Gastric Adenocarcinoma | logistic regression (LR), random forest (RF), support vector machine (SVM), and XGBoost | Yes | Yes | AUC/ AUROC reported | Yes |
| Shen 2024 | Retrospective Study | Colorectal Cancer | LASSO Regression | Yes | Yes | AUC/ AUROC reported | Yes |
| Shi 2012 | Prospective Cohort Study | Breast Cancer | Artificial neural network (ANN): specifically, multilayer perceptron (MLP) network | Yes | Yes | AUC/ AUROC reported | Yes |
| Shi 2024 | Retrospective Study | Metastatic Spinal Tumors | XGBoosting Machine  Logistic Regression  K-Nearest Neighbor  Support Vector Machine  Decision Tree  Random Forest | Yes | Yes | AUC/ AUROC reported | Yes |
| Spelt 2013 | Retrospective Study | Colorectal Cancer with Liver Metastasis | Artifical Neural Network-Effective time dependent hazard ratios (HR) for the risk factors; Harrell's C-Index to test performance | No | No | No | Yes |
| Sun 2023 | Retrospective Study | Hepatocellular Carcinoma | Adaboost | Yes | Yes | AUC/ AUROC reported | Yes |
| vandeBeld 2024* | Retrospective Study | Esophageal Cancer | ResNet18 architecture; long short term memory (LSTM) units; Multimodal Deep Learning Models; Logistic Regression | Yes | Yes | AUC/ AUROC reported | Yes |
| vanNiftrikCHB 2019* | Retrospective Study | Cranial Tumor | Random Forest, Neural Network, Support Vector, Machine and Gradient Boosting | Yes | Yes | AUC/ AUROC reported | No |
| Verma 2023* | Retrospective Study | Pancreatic Cancer | eXtreme Gradient Boosting (XGBoost) | No | No | AUC/ AUROC reported | Yes |
| Vesovic 2023* | Prospective Cohort Study | Lung Cancer | Regression metamodel | No | No | No | Yes |
| Wang 2023 (Combined application of inflammation-related biomarkers to predict postoperative complications of rectal cancer patients: a retrospective study by machine learning analysis.)* | Retrospective Study | Rectal Cancer | Logistic regression, vector machines, regression trees, random forest plot | Yes | Yes | AUC/ AUROC reported | Yes |
| Wang 2023 (Data-Driven Assisted Decision Making for Surgical Procedure of Hepatocellular Carcinoma Resection and Prognostic Prediction: Development and Validation of Machine Learning Models) | Retrospective Study | Hepatocellular Carcinoma | Deep neural networks (DNNs), Deep Model of Survival Prediction, Deep Model of Recurrence Prediction | No | No | AUC/ AUROC reported | Yes |
| Wen 2021 | Retrospective Study | Rectal Cancer | Random forest | Yes | Yes | AUC/ AUROC reported | Yes |
| Wu 2022 | Retrospective Study | Breast Cancer | a naïve Bayes model, a k-nearest neighbor (KNN) model, a support vector machine (SVM) model, a logistic regression model, and a multilayer perceptron (MLP) model | Yes | Yes | AUC/ AUROC reported | Yes |
| Xu 2023 | Retrospective Study | Breast Cancer | Logistic regression (LR), eXtreme Gradient Boosting (XGBoost), k-Nearest Neighbor (KNN), and Support Vector Machine (SVM) | Yes | Yes | AUC/ AUROC reported | Yes |
| Ying 2021* | Retrospective Study | Bladder Cancer | Convolutional Neural Network | No | No | No | No |
| Zaver 2023* | Retrospective Study | Liver Failure (requiring transplantation) | Convolutional neural network-based algorithm | Yes | Yes | AUC/ AUROC reported | Yes |
| Zeng 2023 | Retrospective Study | Breast Cancer | Three AI algorithms: SVM,  XGBoost, and LSTM | No | No | AUC/ AUROC reported | Yes |
| Zeng 2024 | Retrospective Study | Gastric Cancer | DeepSurv Neural Network | Yes | Yes | AUC/ AUROC reported | Yes |
| Zhang 2023 | Retrospective Study | Medullary Thyroid Cancer | Random forest classifier (RFC), gradient boosting decision tree (GBDT), logistic regression (LR), and support vector machine (SVM) | No | Yes | AUC/ AUROC reported | Yes |
| Zhang 2024 (Development and comparison of machine-learning models for predicting prolonged postoperative length of stay in lung cancer patients following video-assisted thoracoscopic surgery.) | Retrospective Study | Lung Cancer | XGBoost, random forest, neural networks | No | No | AUC/ AUROC reported | Yes |
| Zhang 2024 (Deep learning nomogram for preoperative distinction between Xanthogranulomatous cholecystitis and gallbladder carcinoma: A novel approach for surgical decision) | Retrospective Study | Gallbladder Carcinoma | FConv-ResNet18 (Fourier transform-based ResNet model) | Yes | Yes | AUC/ AUROC reported | Yes |
| Zhao 2023 | Retrospective Study | Thyroid Cancer | LASSO/nomogram prediction model | No | No | ROC plot but no AUC value | Yes |
| Zheng 2023 | Retrospective Study | Gastric Cancer | LASSO Cox Regression | Yes | Yes | AUC/ AUROC reported | Yes |
